# Adapting the Cornell assessment of pediatric delirium for Swedish context: translation, cultural validation and inter-rater reliability

**DOI:** 10.1186/s12887-024-04886-w

**Published:** 2024-06-26

**Authors:** Sara Åkerman, Anna Axelin, Chani Traube, Robert Frithiof, Ylva Thernström Blomqvist

**Affiliations:** 1 Department of Surgical Sciences, Anaesthesiology and Intensive Care, Uppsala University, Uppsala, Sweden; 2 Uppsala Centre for Paediatric Anaesthesia and Intensive Care Research, Uppsala, Sweden; 3 Department of Women’s and Children’s Health, Uppsala University, Uppsala, Sweden; 4 Department of Nursing Science, University of Turku, Turku, Finland; 5 Department of Pediatrics, Weill Cornell Medical Center, New York, NY USA

**Keywords:** Pediatric, Delirium, Pediatric intensive care (PICU), Critical care, Sweden, Reliability, CAPD

## Abstract

**Background:**

Pediatric delirium causes prolonged hospital stays, increased costs, and distress for children and caregivers. Currently, there is no delirium screening tool available in Sweden that has been translated, culturally validated, and tested for reliability. This study aimed to translate, culturally adapt, and assess the suitability of the Cornell Assessment of Pediatric Delirium (CAPD) for implementation in Swedish healthcare settings.

**Methods:**

The CAPD was translated and culturally adapted to Swedish context following the ten-step process recommended by the International Society for Pharmacoeconomics and Outcomes Task Force for Translation and Cultural Adaptation. The Swedish CAPD was tested in the pediatric intensive care unit of Uppsala University Hospital, a tertiary hospital in Sweden. Inter-rater reliability was tested using intraclass correlation coefficient (ICC), with both Registered Nurses (RNs) and Assistant Nurses (ANs) conducting parallel measurements using the Swedish CAPD. A reliability score of ICC > 0.75 was considered indicative of good reliability.

**Results:**

After translation of the CAPD into Swedish, 10 RNs participated in the cultural adaptation process. Issues related to word choice, education, and instructions were addressed. Wording improvements were made to ensure accurate interpretation. Supplementary training sessions were organized to strengthen users’ proficiency with the Swedish CAPD. Additional instructions were provided to enhance clarity and usability. Inter-rater reliability testing resulted in an ICC of 0.857 (95% CI: 0.708–0.930), indicating good reliability.

**Conclusion:**

This study successfully translated and culturally adapted the CAPD to align with Swedish contextual parameters. The resulting Swedish CAPD demonstrated good inter-rater reliability, establishing its viability as a tool for measuring delirium among pediatric patients in Swedish pediatric intensive care units.

**Trail registration:**

Not applicable.

**Supplementary Information:**

The online version contains supplementary material available at 10.1186/s12887-024-04886-w.

## Background

Delirium, an acute disturbance of brain function affecting attention, awareness, and cognition [1], can impact children of all ages, from newborns to 18-year-olds [2, 3]. Its varied presentation makes identification challenging, particularly in pediatric intensive care unit (PICU) settings where children are at heightened risk. Risk factors for pediatric delirium (PD) include drug use (e.g., benzodiazepines, anticholinergic medications), mechanical ventilation, young age (< 2 years), developmental delays, and pre-existing anxiety [2, 4, 5].

The consequences of PD are significant, including prolonged mechanical ventilation, extended hospital stays, and increased distress for both children and families [4, 5]. Post-discharge effects can include diminished quality of life and sleep disturbances [6–8], cognitive decline, and higher rates of hospital readmission within a year compared to children without delirium during their hospital stay [9].

Diagnosis of PD is established using the DSMV criteria [10]. Several delirium assessment tools exist, such as the Pediatric Anesthesia Emergence Delirium Scale (PAED) [11], pediatric and pre-school Confusion Assessment Method for the ICU (p/psCAM-ICU) [12, 13], and the Sophia Observation Withdrawal Symptoms-Pediatric Delirium (SOS-PD) [14, 15]. The most commonly used delirium assessment tool is the Cornell Assessment of Pediatric Delirium (CAPD) [3, 16], recommended by the European Society of Pediatric and Neonatal Intensive Care (ESPNIC) and the Society of Critical Care Medicine (SCCM) for daily use in PICUs [17, 18]. The CAPD includes eight questions assessing various aspects of behavior, with a cutoff score ≥ 9 indicating delirium presence. Anchor points are provided for preverbal children to aid in assessment [19, 20]. CAPD has been tested to ensure validity and reliability [21, 22] and also translated into several different languages and cultures [23–29].

Currently, the only available PD measurement tool in Swedish is the SOS-PD. However, to the authors’ knowledge, the translation process of SOS-PD was not reported, and the translated version has not undergone reliability testing. Additionally, SOS-PD is not suitable for all age groups. Therefore, the aim of this study was adapt the CAPD into Swedish and assess its reliability, in order to provide Swedish PICUs with a feasible and efficient tool to use for delirium assessment in children of all ages.

## Methods

**Fig. 1:**
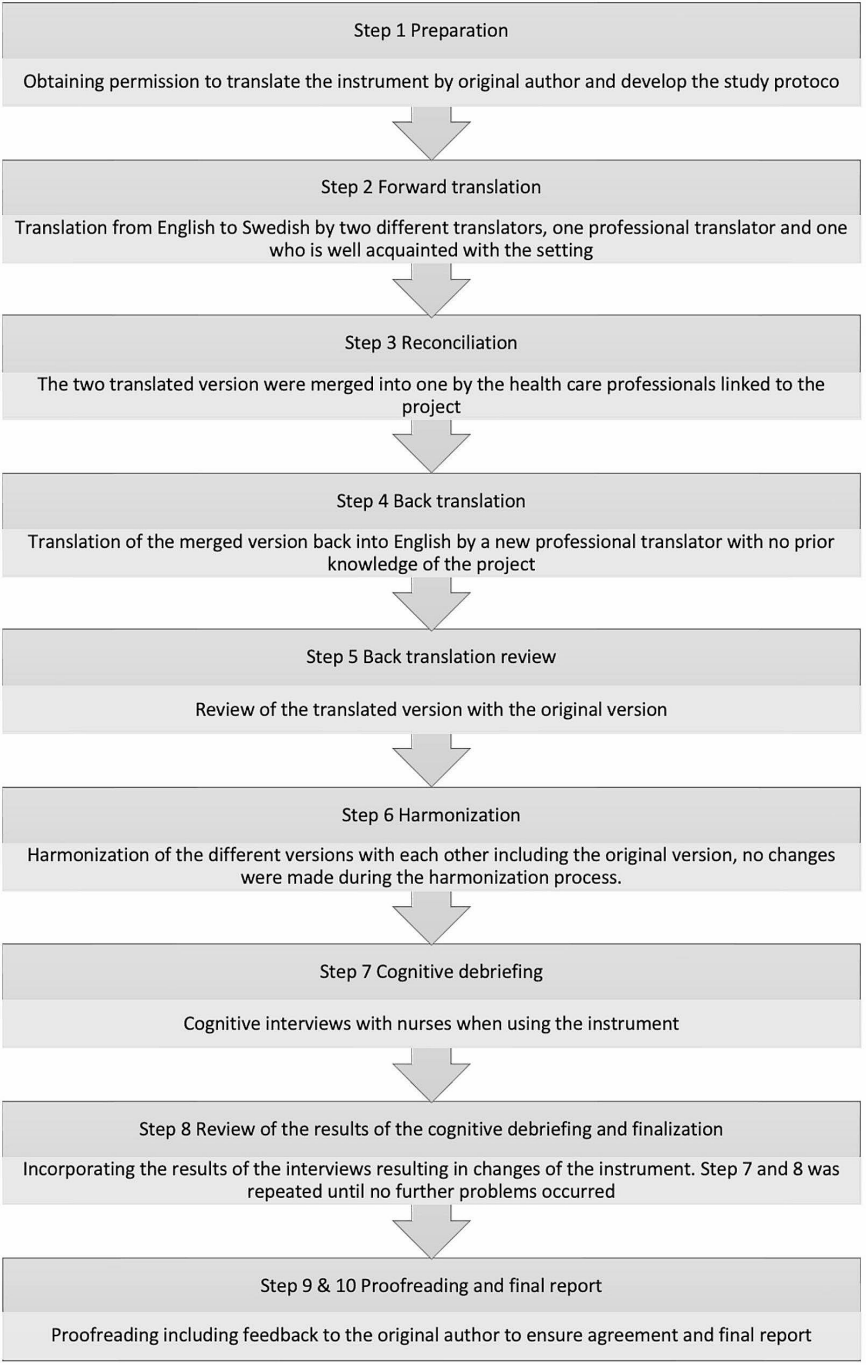
Ten step process by ISPOR task force for translation and cultural adaptation

The translation and adaptation of the CAPD to Swedish context followed the ten steps recommended by the International Society for Pharmacoeconomics and Outcomes (ISPOR) Task Force for Translation and Cultural Adaptation [30] (see Fig. 1).

### Translation

#### Procedure and sampling

Following permission from the original author, the translation of CAPD involved two translators: one professional and the main author, who was familiar with the measurement tool context. Their translations were consolidated through discussions with RNs and physicians knowledgeable about pediatric care and delirium. The merged Swedish version was subsequently back-translated into English by a new translator unfamiliar with the original. A harmonization process ensured consistency across versions. All translations were compared to verify accuracy, and the final version was submitted to the original author for approval.

### Cultural adaptation

To ensure the comprehensibility of the translated CAPD in the Swedish setting, a process of cultural adaptation was conducted using cognitive interviews, a method for testing questionnaires in healthcare settings, following the method outlined by Andersen et al. [31]. This method aims to clarify how respondents understand and interpret different components of healthcare questionnaires.

#### Procedure and sampling

The method, as detailed by Willis [32], typically involves two main components: ‘thinking aloud’ and ‘probing’. During ‘thinking aloud’, the respondent verbalizes thoughts while completing the CAPD questionnaire, while ‘probing’ prompts the interviewer to seek clarifications on the respondent’s understanding of questions, phrases, or their decision-making process. This approach aims to identify any comprehension issues and continues until all problems are addressed. Any changes resulting from the interviews require retesting using the same procedure.

To facilitate structured cognitive interviews, a guide was developed (Additional file 1) and reviewed by the authors. After a practice section (for respondents to become familiar with the ‘think aloud’ method, understand the CAPD tool, and seek clarification if necessary), respondents utilized the CAPD while explaining their thought process. Following this, both respondent and interviewer engaged in a discussion, covering predefined probing questions, to address each aspect of the CAPD. The guide concluded by inviting respondents to offer additional insights or comments regarding the CAPD.

Inclusion criteria for RNs participating in the cultural adaptation process were having worked in a PICU for more than one year. The RNs all received a brief lecture on pediatric delirium and study details. All participants received verbal and written information, with informed consent obtained. The entire interview, led by the corresponding author who possessed sufficient training and familiarity with the setting, was audio recorded. The introduction and probing phases took place in a private room, while the ‘think aloud’ component occurred at the bedside.

#### Data analysis

A pre-designed matrix, following Willis [32], was utilized to analyze the interviews. The matrix consisted of eight problem categories; (1) wording, (2) instructions, (3) clarity, (4) assumptions, (5) knowledge/memory, (6) sensitive questions, (7) response categories, or (8) other. They were subsequently divided into five possible solution categories; (A) Change the wording or the complete phrase, (B) Clarify the purpose of the question or the words used, (C) Education, (D) Changing/adding to the physical design or (E) Unresolved due to original design.

### Testing the reliability

To test interrater reliability, assistant nurses (ANs) (responsible for providing care to the child alongside the RN during a shift) were included in the study. The process of education and obtaining informed consent for the ANs followed the same procedures as previously described for the RNs.

#### Procedure

Using convenience sampling, the main author identified a suitable child for delirium assessment. Children were eligible for inclusion if they were 0–18 years old and arousable to verbal stimulation. Children were deliberately sampled across the age spectrum. The main author ensured that each trio (RN, AN, and child) was unique. The CAPD was then scored by one RN and one AN, who had both cared for the child during their shift. The RN and AN were instructed that the CAPD should be completed by the end of the shift, each receiving a paper copy to fill out independently. They were explicitly instructed not to discuss or share any information about scoring. The results of the measurements were not disclosed between the assessors.

#### Data analysis

To test reliability of CAPD, inter-rater reliability using ICC was utilized. ICC is commonly used in clinical settings and measures the consistency of measurements made by different raters, indicating how similar these measurements are when assessing the same subject. A two-way random effects, single rater ICC model was employed to examine consistency [33]. In this model, raters, such as RNs and ANs working in a PICU, are considered as a random sample from a larger population with similar characteristics. The ICC values were interpreted as indicated by Koo and Li (2016) : ICC < 0.5 indicated poor reliability, ICC between 0.5 and 0.75 indicated moderate reliability, ICC between 0.76 and 0.90 indicated good reliability, and ICC > 0.90 indicated excellent reliability. The ICC method for assessing reliability has been employed similarly in previous studies evaluating the reliability of the CAPD [24, 34]. Additionally, a Bland-Altman plot was included to enhance understanding of inter-rater reliability.

## Result

### Translation

The translation process was conducted as outlined in the method section, with adjustments made at each step, which can be referenced in Additional file 2. These modifications primarily centered on the selection of wording, to better align with Swedish healthcare terminology and usage.

### Cultural adaptation

A total of ten ICU RN (Table 1) participated in the interviews and assessed a total of nine children (Table 2).

**Table 1:** Interviewed registered nurses

Nurses (*n* = 10)	Round 1 (*n* = 6)	Round 2 (*n* = 4)
Age, years median (IQR)	37 (29.5–39.2)	37 (27.5–56.2)
Work experience as RN, years median (IQR)	11.5 (7.5–15.8)	14 (6.3–31.5)
Work experience as ICU RN, years median (IQR)	3 (2–4)	5.6 (2.4–18.5)

**Table 2:** Children that were analyzed using CAPD during the interview process

Children (*n* = 9)	Round 1 (*n* = 5)	Round 2 (*n* = 4)
Age, years median (IQR)	0.8 (0.3–6.7)	0.9 (0.3–9.2)
Sedating/pain medication (n)	4	2
Postoperative care (n)	3	2

Interviews were conducted in two rounds. In the first round, six RNs participated, assessing a total of five children. These interviews were analyzed by the corresponding author in consultation with the other authors, to identify issues in the translated version of the CAPD. Cognitive interviews demonstrated that the RNs understood most CAPD elements. For examples, when interviewees were queried about elucidating ‘if the child is restless’, one respondent phrased it as follows:

…these little movements of the hands and feet, lying down and twitching, tongue licking the lips, or throwing themselves back and forth….

When asked how to explain the word ‘inconsolable’, one respondent said:

…nothing can calm the child, whatever we do nothing happens, we have tried painkiller and nothing happens, the parents have picked [the child] up but nothing happens, we have tried giving food but nothing happens….

The interpretation of ‘react to interaction’ was expressed as follows:

…that it responds to what I do and, above all, is unhappy when I take a blood pressure or temperature, a [small] healthy child is unhappy with us and we want to see that!

**Fig. 2:**
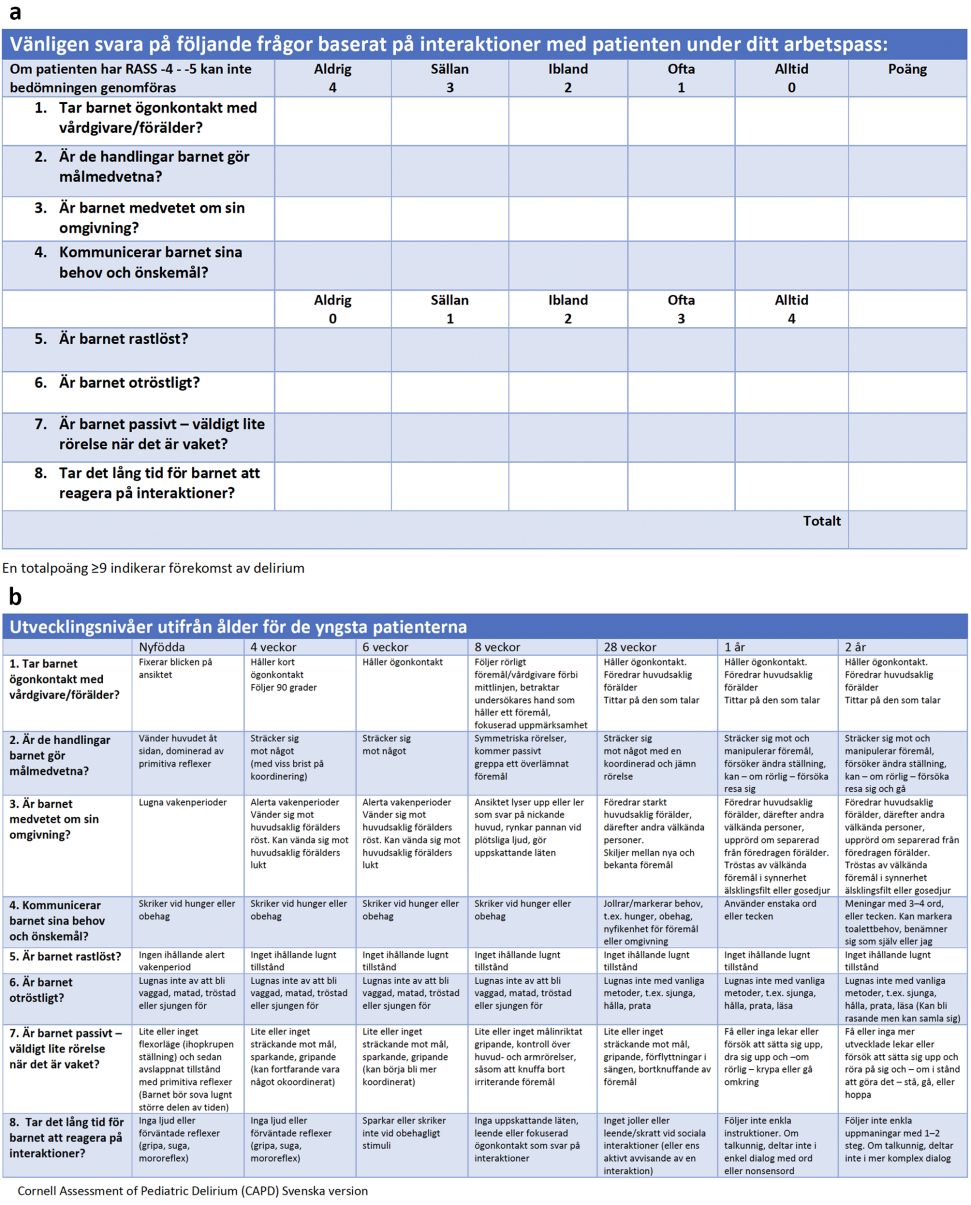
(**a**) Translated version of Cornell assessment of pediatric delirium. (**b**) Translated version of anchor points for Cornell assessment of pediatric delirium

However, several issues were identified during the cognitive interviews as delineated in Table 3. Changes were implemented, and new interviews were conducted to assess whether additional issues arose after the adjustments. After four additional interviews, no further issues were identified. The interviews collectively lasted for 316 min, ranging from 19 to 48 min each. The final Swedish version of CAPD and associated anchorpoints (Fig. 2a and b) were sent to the original author for approval.

**Table 3:** Problems and solutions from the cultural adaptation process

Description of issue	Solution
**Problem category 1) Wording:**	**A) Change the wording or the complete phrase:**
- Different interpretations of the word ‘action’	- the word ‘actions’ changed to ‘act’
- The use of the word ‘strong’ doesn’t fit the context	- ‘strong’ was replaced with ‘sudden
- Not reading the word ‘little’ in the combination ‘very little’	- a revision to ensure it was interpreted correctly as ‘little
**Problem category 2) Instruction**	**B) Clarify the purpose of the question or the words used**:
- Lacks instructions on when assessment is not possible (i.e.: children who are not arousable to verbal stimulation)	- Information about situations where assessment is not possible was added
- When should the assessment take place?	- Specifying that the assessment should take place at the end of the shift
- Whose interactions are the questions aimed at - What actions should be considered in the assessment	- Clarifications were made that the assessment pertains to actions carried out by the child
**Problem category 3) Clarity**	**B) Clarify the purpose of the question or the words used**:
- Who is the primary caregiver, can mean parents, caregivers, health professionals simultaneously or exclusively of each other	- Clarified the interpretation of ‘primary caregiver’ by adjusting the wording to include both caregiver and parent
- The use of the phrase ‘long time’ is vague and therefore difficult to assess	- Changed ‘does it take a long time for the child to react to interaction’ to ‘does it take longer time for the child to react to interaction’
- Unclear what ‘calling himself or herself or me’ means	- Modified ‘calls him/herself or me’ to ‘refers to him/herself or me’
**Problem category 4) Assumptions**	**A) Change the order of the words or the wording** :
- Assumes that actions must occur	- Clarified which interactions the specific questions addressed by adjusting wording
- Assumes that the interactions have to be made with the nurse	- Changed ‘are the child’s actions purposeful’ to ‘are the actions the child takes purposeful’ for clarity
**Problem category 5) Education and training**	**C) Education**:
- Not enough knowledge about the child to carry out the assessment correctly - Difficulty in selecting the age category due to premature birth, developmental disability or being between age classifications.	- Further education on using the assessment tool was provided, e.g. age classification, how to think regarding developmental disability.
- Misunderstanding of the phrase ‘passive grasping’ - Unaware of the phrase ‘flexed state’	- Education was also given to clarify misconceptions surrounding terms like ‘passive grasping’ and ‘flexed state’
- Not able to distinguish between delirium/pain/withdrawal	- Additional educational efforts were made to differentiate between delirium, pain, and withdrawal
**Problem category 7) Response category**:	**D) Unresolved due to original design**
- Difficult to distinguish between ‘rarely’ and ‘sometimes’ when choosing a response category	-
**Problem category 8) Other**:	**E) Changing/adding to the physical design**:
- Lack of information about the cut-off score for delirium. - Problems with the physical design: Instructions were not clear enough	- Resolving physical design issues involved incorporating details about the cut-off score and enhancing instruction clarity.

The table provides an overview of the issues encountered during the cultural adaptation process, along with their corresponding solutions. Each problem category is numbered 1–8, and the solutions are denoted by letters A-E

### Testing the reliability

A total of 19 RNs and 15 ANs assessed 18 children using the Swedish CAPD (see Tables 4 and 5). A total of 32 duplicate assessments using the Swedish CAPD were completed.

**Table 4:** Characteristics of RNs and ANs in reliability testing

	*N*	Age, years median (IQR)	Work experience as RN/AN, years median (IQR)	Work experience as ICU RN/AN years median (IQR)
RN	19	38.8 (28.75–46.5)	12.9 (5-17.5)	5.8 (2-7.5)
AN	15	43.3 (35–52)	17.9 (9-25)	6.3 (2.5-9)

**Table 5:** Characteristics of children assessed with CAPD during reliability testing

Children (*n* = 18)	
Age, months, median (IQR, min, max)	58.8 (10.8–114, 0.3, 168)
Respiratory support (n) (invasive or non-invasive ventilation)	7
Developmental disability (n)	8
Reason for admission (n)	Respiratory failure (6) Encephalitis (3) Postoperative care (3) Burn injury (2) Ventricular shunt dysfunction (2) Seizures (1) Ketoacidosis (1)

**Fig. 3:**
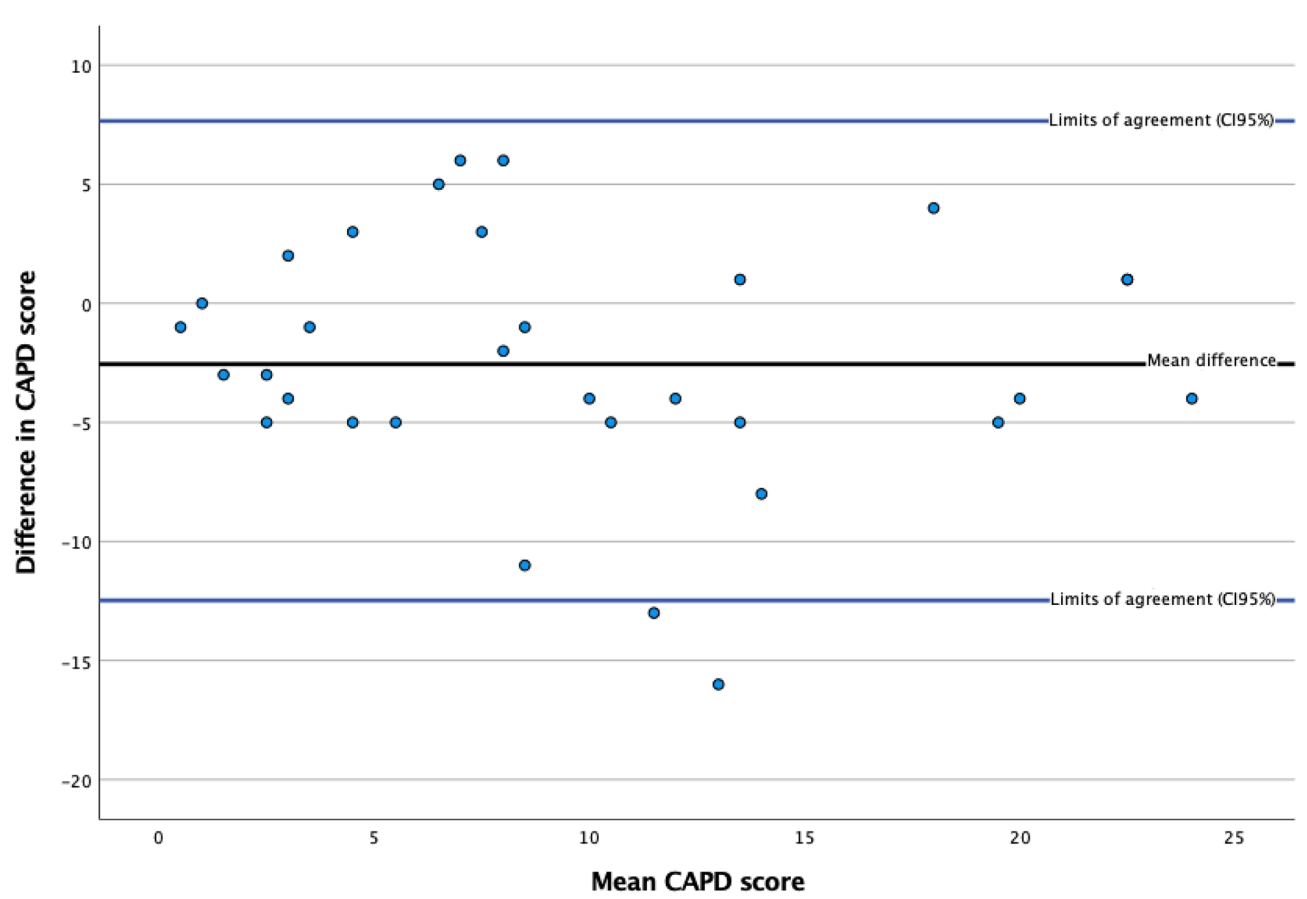
Bland-Altman plot for all measurements

The overall reliability of the Swedish CAPD was good, with an ICC of 0.857 (95% CI: 0.708–0.930). Logistic regression of the Bland-Altman plot, created to visualize score differences against score averages, showed no significance (*P* = 0.555), indicating the absence of proportional biases (see Fig. 3).

**Fig. 4:**
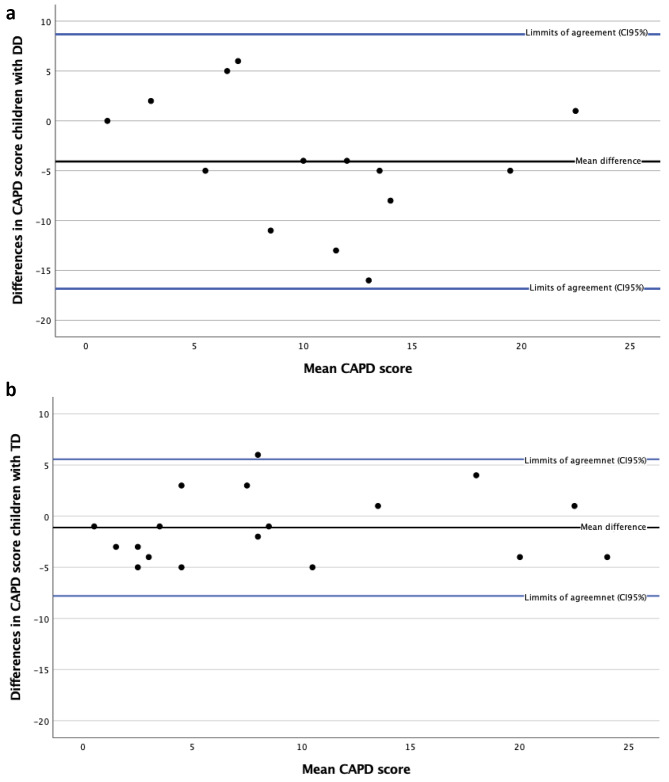
(**a**) Bland-Altman plot for children with developmental disability. (**b**) Bland-Altman plot for children with typical development

In an exploratory sub-group analysis, the CAPDs were divided into two groups: assessments in children with typical development (TD, *n* = 18), and children with developmental disability (DD). (DD was defined as a pre-existing disability that required support from health care services prior to hospitalization). When calculating ICC for the two groups, CAPDs in children with TD resulted in an ICC of 0.948 (CI: 0.862–0.981), indicating excellent reliability. CAPDs in children with DD resulted in an ICC of 0.700 (CI: 0.067–0.904), indicating only moderate reliability. The Bland-Altman plots also showed that the group with DD had a greater dispersion compared to the group without developmental disability (Fig. 4a and b).

## Discussion

Given the potential severity of delirium consequences, both during hospitalization [4, 5, 35], and post-discharge [6–9] it is imperative for children in a PICU to be assessed for delirium using a valid and reliable delirium assessment tool [17, 18]. Given the fluctuating nature of delirium symptoms, conducting multiple assessments over the child’s hospitalization enhances the likelihood of detecting delirium [2, 4, 35]. Therefore, the importance of a user-friendly and efficient delirium assessment tool cannot be overstated. In addition, longitudinal assessments enable detection of response to interventions over time, including early rehabilitation [36], implementation of A-F bundles [37], non-pharmacological [38], and/or multi-disciplinary interventions [39]. This may lead to decreased delirium and improvements in outcomes for affected children [40, 41].

In this study, we have successfully translated and culturally adapted the CAPD for Swedish context. The cultural adaptation process provided valuable insights that allowed for further improvement in the final Swedish CAPD. We recommend that this rigorous approach be used when translating the CAPD into other languages and cultures.

Reliability testing of the Swedish CAPD has established good interrater reliability. This is similar to results in other settings and languages which also demonstrated good interrater reliability [23–25]. However, it’s important to note that this was a single-center, small-scale study. Larger-scale, preferably multicenter studies may be necessary to further evaluate interrater reliability, especially in the assessment of the youngest children, where implementation can be more challenging [22, 42, 43].

Another challenge arises when measuring delirium in children with developmental disabilities [44, 45], In our cohort, the Swedish CAPD maintained moderate reliability in children with DD. However, the results from this subgroup analysis must be interpreted with extra caution due to the very small sample size. In other studies, the CAPD was used in conjunction with the Richmond Agitation Sedation Scale (RASS), which dramatically improved specificity in this hard-to-assess population [46, 47]. Future studies should consider investigating whether the interrater reliability of the Swedish CAPD can be improved when coupled with the RASS in children with underlying DD.

Strengths of this study include the meticulous cultural adaptation process and the strong collaboration with the original authors of the CAPD. However, important limitations exist. Most significantly, this is a single-center study which may limit generalizability to all Swedish PICUs. Involvement of multiple PICUs during the cultural adaptation may have improved the final version. This approach has been employed in other studies where multiple sites collaborated to translate measurement instruments for pain in premature infants [31]. In addition, future studies examining the psychometric properties and interrater reliability of the Swedish CAPD with larger sample sizes and in diverse settings may further establish feasibility for use in Swedish PICUs nationwide.

## Conclusion

This study has translated and culturally adapted the CAPD to Swedish context and established its inter-rater reliability. The Swedish CAPD is a valuable tool for assessing delirium presence among critically ill children in Sweden.

### Electronic supplementary material

Below is the link to the electronic supplementary material.

**Supplementary Material 1: Additional file 1:** Interview guide Nurses. The interview guide utilized during the cognitive interviews for the cultural adaptation process

**Supplementary Material 2: Additional file 2:** All versions of CAPD during the translation and cultural adaptation process. Each version displayed from the initial translations to the altered version of CAPD after the first round of cognitive interviews

**Supplementary Material 3: Additional file 3:** COREQ Checklist. The COREQ (Consolidated Criteria for Reporting Qualitative Research) checklist is designed to ensure comprehensive reporting of qualitative research studies

## Data Availability

The datasets used and/or analyzed during the current study are available from the corresponding author on reasonable request.

## Abbreviations

AN: Assistant Nurses
CAPD: Cornell Assessments of Pediatric Delirium
ESPNIC: European Society of Pediatric and Neonatal Intensive Care
ICC: Interclass Correlation Coefficient
ICU: Intensive Care Unit
ISPOR: International Society for Pharmacoeconomics and Outcomes
PD: Pediatric Delirium
PICU: Pediatric Intensive Care Unit
RASS: Richmond Agitation Sedation Scale
RN: Registered Nurses
SCCM: Society of Critical Care Medicine
