# Supplementary material for: Adapting the Cornell assessment of pediatric delirium for Swedish context: translation, cultural validation and inter-rater reliability
Source: BMC Pediatr. 2024 Jun 26;24:413. doi: 10.1186/s12887-024-04886-w (PMC11202322; doi:10.1186/s12887-024-04886-w)
Supplement: Supplementary file 1 — Supplementary Material 1: Additional file 1: Interview guide Nurses. The interview guide utilized during the cognitive interviews for the cultural adaptation process [file 12887_2024_4886_MOESM1_ESM.pdf]

Additional file 1: Interview guide Nurses

CAPD INTERVIEW GUIDE FOR NURSES

INTRODUCTION

Thank you for agreeing to participate in this study. The purpose of the interview is to obtain your views on the newly translated Cornell Assessment for Pediatric Delirium (CAPD) instrument, which identifies and measures the prevalence of delirium in children in intensive care. The aim of the interviews is to find ambiguities and difficulties with the translated instrument.

Participation is voluntary and you can choose to stop the interview at any time without giving a reason. There are no known risks involved in participating and the interview will last approximately one hour.

The interview will be recorded with your consent and notes will be taken during the interview. All material will be de-identified and coded and stored in such a way that no unauthorized persons have access to it. The information provided by you will not enable identification. The information obtained from the interviews will be compiled and published in a scientific journal.

THINK ALOUD
You will first read about the CAPD and then try to use it practically. While you are assessing your child using the CAPD, I will ask you to think aloud. This means that I will not ask you any questions while you are assessing your child, but rather ask you to put into words your thoughts and how you perceive and interpret the different parts of the instrument, how easy or difficult it is to understand them and how easy or difficult it is to use the scale.<br><br>To try this out, we will carry out a small exercise:<br>*Imagine you are in your home, I want you to tell me how many windows you have in your home. As you count the windows, I want you to tell me what you see and what you think about.*<br><br>Now I will let you look at the assessment tool and familiarize yourself with it. I want you to read through the whole assessment tool and think aloud as you do so, just as we did during the exercise. Tell me what you are thinking, if there are any questions or concerns as you go along. Let me know when you feel done.<br><br>Now you can try using the CAPD in practice. We will visit a patient who has previously given his/her consent to participate in the study, either by him/herself or via a guardian. You have previously met the patient and will use what you see now and what you know previously to make your assessment of the presence of delirium using the CAPD. As you make your assessment, I want you to think out loud as we just practiced. There is no "right" answer and I cannot help you in the meantime. What is important is that you think out loud about how you are making your assessment when filling in the instrument, not what answer you choose or what the score is.<br><br>Do you have any questions?<br><br>Then we go to the patient and remember to try to think out loud as much as you can while doing the assessment.

Additional file 1: Interview guide Nurses

VERBAL PROBING
We have now left the patient and are *sitting in a separate room.*<br><br>You have now completed your assessment using the CAPD. I will now ask you some questions about how you perceived the assessment instrument. Remember, there is no right or wrong, I want to know how you perceive the instrument, the wording and the choice of words.
Cross-cutting issues/Probes
• How do you perceive the question?<br>• Can you repeat the question in your own words?<br>• How well is the question adapted to your situation? Can you tell us more?<br>• How do you think when you answer?
Follow-up questions
• Can you tell us more about it?<br>• How easy or difficult is it for you to answer this question?<br>• What do you think about when you try to answer the question (if the person cannot)?<br>• You seem a bit unsure, if so, can you tell us why?<br>• What made you change your answer?<br>• Can you tell me what you thought when I asked "xxx"?<br>• You seem to find it difficult to understand what is meant, do you have any suggestions on how to make "xxx" more understandable?<br>• Would you have chosen any other word?
Scoring
• How easy or difficult was it for you to choose the points? Why?<br>• How easy or difficult was it for you to distinguish between the different options? Why?<br>• How did you choose points?<br>• Can you tell us more about it?<br>• How easy or difficult was it for you to select points from the examples given in the different sections? Why?<br>• How easy or difficult was it for you to remember? Can you tell me more?

Additional file 1: Interview guide Nurses

Part 1: CAPD		
a)	**The instructions**<br>"*Please answer the following questions based on your interaction with the patient during your shift:*"	How do you interpret the initial instruction?<br>Can you repeat it in your own words?<br>How well is the question adapted to your situation? Can you tell us more?<br>How do you interpret the word 'interaction' in this context?<br>What does the phrase "during your shift" mean?
	The questions	
b)	1. Does the child make eye contact with the caregiver?	Throughout the instrument we have chosen to use the word "carer", can you explain in your own words what is meant?<br>How do you interpret the word 'carer'?
c)	2. Are the child's actions purposeful?	How do you interpret the word "actions"?<br>What does 'purposeful' mean in this context?
d)	3. Is the child aware of their environment?	In your own words, how would you explain what is meant by "aware of your surroundings"?
e)	4. Does the child communicate their needs and wishes?	What do the words "needs" and "wants" mean in this question?<br>Would you have chosen any other words?
f)	5. Is the child restless?	What does the word 'restless' mean to you in this context?
g)	6. Is the child inconsolable?	What does the word 'inconsolable' mean to you in this context?
h)	7. Is the child passive - very little movement when awake?	In your own words, can you describe what "is the child passive" means to you?<br>How do you interpret the word 'passive'?
i)	8. Does the child take a long time to respond to interactions?	In this question, what does the phrase "it takes a long time" mean to you?<br>How do you interpret the phrase "respond to interaction"?
j)	**The scoring system:**	How easy or difficult was it for you to choose the points? Why?<br>How easy or difficult was it for you to distinguish between the different options? Why?<br>After four questions the score changes, how easy or difficult was it for you to score after that? Why?<br>How easy or difficult was it for you to compile the scores? Why?

Part 2: Assessment points	
**k) Title** *"Developmental levels by age for the youngest patients"*	How do you interpret the word "levels of development" in this context? Would you have chosen another word?<br>Can you formulate this sentence in your own words?
**l) Age classification**	How easy or difficult is it for you to choose the age category based on the child you have assessed? Can you tell us more?<br>Would you have liked more instructions?
**m) Scoring** Asked after each question	How did you choose points?<br>Can you tell us more about it?<br>How easy or difficult was it for you to select points from the examples given in the different sections? Why?<br>How easy or difficult was it for you to remember? Can you tell me more?
**n)** 1. Does the child make eye contact with the caregiver?	**NF:** Can you describe in your own words what it means to "fix your eyes on your face"?<br>**4v:** Can you describe in your own words what "making brief eye contact" means? In this sentence, how do you interpret "follows 90 degrees"?<br>**6v:** Can you describe in your own words what "keeping eye contact" means?<br>**8v:** What does 'following a moving object' mean to you? What does the word "centerline" mean to you in this sentence? How easy or difficult was it for you to understand the phrase "watching the examiner's hand holding an object"? Why? What does the phrase "focused attention" mean when used in this sentence?<br>**28v:** Can you describe in your own words what "keeping eye contact" means? What does "preferred primary caregiver" mean when used in this context?<br>**1y:** Can you describe in your own words what "keeping eye contact" means? What does "preferred primary caregiver" mean when used in this context?<br>**2y:** Can you describe in your own words what "keeping eye contact" means? What does "preferred primary caregiver" mean when used in this context?
**o)** 2. Are the child's actions purposeful?	**NF:** In this phrase, what does it mean to "turn your head to the side"? How do you interpret the word "dominated"? What does the phrase "primitive reflexes" mean to you"?<br>**4v:** How easy or difficult is it for you to respond to the phrase "reaches towards something"? Why? What does "some lack of coordination" mean to you in this context?<br>**6v:** How easy or difficult is it for you to respond to the phrase "reaches towards something"? Why?<br>**8v:** Can you describe in your own words what "symmetrical movements" means? How do you interpret the phrase "passive gripping"?<br>**28v:** How easy or difficult is it for you to respond to the phrase "reaches towards something"? Why? What does "coordinated smooth movement" mean to you in this sentence?<br>**1y:** How easy or difficult is it for you to respond to the phrase "reaches towards something"? Why? What does the word "manipulate" mean to you in this context? How easy or difficult was it for you to understand the phrase "tries to change position, can - if mobile - try to get up"? Can you elaborate?<br>**2y:** How easy or difficult is it for you to respond to the phrase "reaches towards something"? Why? What does the word "manipulate" mean to you in this context?

		How easy or difficult was it for you to understand the phrase "tries to change position, can - if mobile - try to get up and walk"? Can you elaborate?
p)	3. Is the child aware of their environment?	**NF:** In this context, what does 'quiet time' mean to you?<br>**4v:** In this context, what does "alert waking periods" mean to you? Can you describe in your own words how you understand the phrase "primary caregiver" in this context? What does the phrase "turn towards" mean in this context?<br>**6v:** In this context, what does "alert waking periods" mean? Can you describe in your own words how you understand the phrase "primary caregiver" in this context? What does the phrase "turn towards" mean in this context?<br>**8v:** How do you interpret the phrase "the face lights up" in this context? Can you describe in your own words what "frowning" means? How do you interpret the phrase "straight sounds" in this context? Can you describe in your own words how you interpret "making appreciative sounds"? Would you have chosen any other wording? (gurgling/cooing?)<br>**28v:** Can you describe in your own words what "strongly prefer" means in this context? What does the phrase "well-known people" mean? How easy or difficult was it for you to consider the phrase "distinguishes between new and familiar objects"? Why?<br>**1y:** What does the phrase "well-known people" mean to you? What does 'separated' mean in this context? Can you describe in your own words what is meant by "comforted by familiar objects"?<br>**2y:** What does the phrase "well-known people" mean to you? What does 'separated' mean in this context? Can you describe in your own words what is meant by "comforted by familiar objects"?
q)	4. Does the child communicate their needs and wishes?	**NF:** What does 'screaming' mean in this context? How do you interpret the word "discomfort"?<br>**4v:** What does 'screaming' mean in this context? How do you interpret the word 'discomfort'?<br>**6v:** What does 'screaming' mean in this context? How do you interpret the word 'discomfort'?<br>**8v:** What does 'screaming' mean in this context? How do you interpret the word "discomfort"?<br>**28v:** When used in this sentence, what is the meaning of 'jolting/marking needs'? The suggestions listed "hunger, discomfort, curiosity about objects or surroundings" how easy or difficult are they to respond to? Can you tell us more?<br>**1y:** What does the phrase "single word" mean in this context? Can you describe in your own words what "sign" means in this context?<br>**2y: What does** "sign" mean in this context? What is the meaning of "marking toilet needs" in this formulation? Can you describe in your own words what is meant by the phrase "says himself or herself or I"?
r)	5. Is the child restless?	**NF:** In this context, what does 'no sustained period of alertness' mean?<br>**4v:** In this context, what does 'no sustained state of calm' mean?<br>**6v:** In this context, what does 'no sustained state of calm' mean?<br>**8v:** In this context, what does "no sustained state of calm" mean?<br>**28v:** In this context, what does "no sustained state of calm" mean?<br>**1y:** In this context, what does "no sustained state of calm" mean?<br>**2y:** In this context, what does "no sustained state of calm" mean?

**s)**	6. Is the child inconsolable?	**NF:** How do you interpret "caregiver" in this context? How well or badly do the suggestions "rocking, singing, feeding, comforting" fit the context? Would you have chosen any other words?<br>**4v:** How do you interpret "caregiver" in this context? How well or badly do the suggestions given "cradles, sings, feeds, comforts" fit the context? Would you have chosen any other words?<br>**6v:** How do you interpret "caregiver" in this context? How well or badly do the suggestions "rocking, singing, feeding, comforting" fit the context? Would you have chosen any other words?<br>**8v:** How do you interpret "caregiver" in this context? How well or badly do the suggestions "rocking, singing, feeding, comforting" fit the context? Would you have chosen any other words?<br>**28v:** How do you interpret "common methods" in this context? How well or badly do the suggestions given "singing, holding, talking" fit the context? Would you have chosen any other words?<br>**1y:** How do you interpret "common methods" in this context? How well or badly do the suggestions given "singing, holding, talking, reading" fit in the context? Would you have chosen other words?<br>**2y:** How do you interpret "common methods" in this context? How well or badly do the suggestions given "sing, hold, talk, read" fit the context? Would you have chosen other words? What does the phrase "can get furious but can collect themselves" mean to you?
**t)**	7. Is the child passive - very little movement when awake?	**NF:** Can you describe in your own words what is meant by the phrase "little or no flexor position (crouched position)" in this sentence? How do you interpret "relaxed state"? What does "primitive reflexes" mean when used here? Would you have chosen any other wording? How easy or difficult is it for you to take a position based on the examples? Why?<br>**4v:** Can you describe in your own words what is meant by the phrase "little or no reaching for objects, kicking, grabbing" in this sentence? What does "coordinated" mean when used here? How easy or difficult is it for you to take a position based on the examples? Why?<br>**6v:** Can you describe in your own words what is meant by the phrase "little or no reaching for objects, kicking, grabbing" in this sentence? How do you interpret "may start to become more coordinated" when used here? How easy or difficult is it for you to take a position based on the examples? Why?<br>**8v:** How do you interpret "little or nothing" in this context? What does 'purposeful grabbing' mean to you? Can you describe in your own words what is meant by "control of head and arm movements"? How good or bad is the example given of "pushing away annoying objects"? Would you have chosen something else? How easy or difficult is it for you to make a decision based on the examples? Why?<br>**28v:** How do you interpret "little or nothing" in this context? Can you describe in your own words what is meant by "reaching for targets, grabbing"? How do you interpret "moving in bed" and "pushing away objects" in this context? How easy or difficult is it for you to take a position based on the examples? Why?<br>**1y:** How do you interpret "few or none" in this context? How well or badly do the examples given "try to sit up, pull yourself up and - if mobile - crawl or walk around". How easy or difficult is it for you to make a decision based on these examples? Why?

		**2y:** How do you interpret "few or none" in this context? How well or badly do the examples given "developed games or attempts to get up and move around and - if able to do so - stand, walk, or jump". How easy or difficult is it for you to decide on the basis of these examples? Why?
u)	8. Does the child take a long time to respond to interactions?	**NF:** Can you describe in your own words what is meant by "no sounds or anticipatory reflexes"? How good or bad are the examples given "grasping, sucking, mororeflexes"?<br>**4v:** Can you describe in your own words what is meant by "no sounds or anticipatory reflexes"? How good or bad are the examples given "grasping, sucking, mororeflexes"?<br>**6v:** How do you interpret the phrase "kick or scream" when used in this context? Can they describe in their own words what is meant by "unpleasant stimuli"?<br>**8v:** What does 'no appreciative sounds, smiles or focused eye contact' mean in this context? What does 'interaction' mean in this formulation?<br>**28v:** What does "no yelling or smiling/laughing in social interactions" mean in this context? Can you describe in your own words what is meant by the phrase "or even active rejection" How do you interpret the word "interaction" in this context?<br>**1y:** Can they describe in their own words what is meant by "does not follow simple instructions"? How do you interpret the phrase "if articulate, does not engage in simple dialog with words or nonsense words"?<br>**2y:** Can they describe in their own words what is meant by "does not follow simple 1-2 step prompts"? How do you interpret the phrase "if numerate, does not engage in more complex dialog"?
v)	Closure	Is there anything you want to say about the instrument that we haven't talked about? Anything else you want to add?<br><br>Thank you for taking the time to participate in this study, your contribution will help to make this instrument as good as possible!

INFORMATION ABOUT THE NURSE

Finally, I would like to ask some questions about you:

Age: ______________________

Gender: ________________________

Utbildning: ___________________

Clinical years: __________

Clinically active in intensive care: _______________

Previous experience with delirium: _________________

If yes, have you received any training on delirium:

____
