# Supplementary material for: Adapting the Cornell assessment of pediatric delirium for Swedish context: translation, cultural validation and inter-rater reliability
Source: BMC Pediatr. 2024 Jun 26;24:413. doi: 10.1186/s12887-024-04886-w (PMC11202322; doi:10.1186/s12887-024-04886-w)
Supplement: Supplementary file 2 — Supplementary Material 2: Additional file 2: All versions of CAPD during the translation and cultural adaptation process. Each version displayed from the initial translations to the altered version of CAPD after the first round of cognitive interviews [file 12887_2024_4886_MOESM2_ESM.pdf]

Additional file 2: All versions of CAPD during the translation and cultural adaptation process

Step 2: Translated version by translator 1

Vänligen svara på följande frågor baserat på din interaktion med patienten under ditt arbetspass:

	Aldrig<br>4	Sällan<br>3	Ibland<br>2	Ofta<br>1	Alltid<br>0	Poäng
**1. Tar barnet ögonkontakt med vårdaren?**						
**2. Är barnets handlingar målmedvetna?**						
**3. Är barnet medvetet om sin omgivning?**						
**4. Kommunicerar barnet sina behov och önskemål?**						
	**Aldrig<br>0**	**Sällan<br>1**	**Ibland<br>2**	**Ofta<br>3**	**Alltid<br>4**	
**5. Är barnet rastlöst?**						
**6. Är barnet otröstligt?**						
**7. Är barnet passivt – mycket lite rörelse när det är vaket?**						
**8. Dröjer det länge för barnet att reagera på interaktioner?**						
					**Totalt**	

Förankringspunkter för utvecklingen hos de yngsta barnen

	Nyfödda	4 veckor	6 veckor	8 veckor	28 veckor	1 år	2 år
**1. Tar barnet ögonkontakt med vårdgivaren?**	Fixerar på ansiktet	Håller blicken kortvarigt<br><br>Följer med huvudet 90 grader	Håller blicken	Följer föremål /vårdnadsgivare som rör sig förbi mittlinje, uppmärksammar undersökarens hand, kan fokusera	Håller blicken, föredrar primär vårdgivare<br>Tittar på den som talar	Håller blicken, föredrar primär vårdgivare.<br>Tittar på den som talar	Håller blicken, föredrar primär vårdgivare.<br>Tittar på den som talar
**2. Är barnets rörelser målmedvetna?**	Rör huvud från sida till sida, mestadels primitiva reflexer	Sträcker sig efter föremål (delvis o-koordinerat)	Sträcker sig efter föremål	Symmetriska rörelser, kommer passivt att greppa föremål som överlämnas	Sträcker sig koordinerat efter föremål med smidiga rörelser	Sträcker sig efter och manipulerar föremål, försöker ändra position, om rörlig försöker sätta sig upp	Sträcker sig efter och manipulerar föremål, försöker ändra position, om rörlig försöker sätta sig upp och gå
**3. Är barnet medvetet om sin omgivning?**	Tid då barnet är lugn och vaken	Tid då barnet är vaken och alert. Vänder sig mot den primära vårdgivarens röst. Kan vända sig mot lukten av den primära vårdgivaren	Längre tid som vaken och alert. Vänder sig mot den primära vårdgivarens röst. Kan vända sig mot lukten av den primära vårdgivaren	Ansiktet lyser upp eller ler som svar på nickande huvud, rynkar ihop ansiktet vid starka ljud, gör läten	Föredrar mamma/primär vårdgivare starkt, efter det andra bekanta<br>Särskiljer på nya och bekanta föremål	Föredrar primär vårdgivare, efter det andra bekanta, blir upprörd vid separation från den föredragna vårdgivaren.<br>Blir tröstad av kända föremål, framför allt favoritfilt eller gosedjur	Föredrar primär vårdgivare, efter det andra bekanta, blir upprörd vid separation från den föredragna vårdgivaren.<br>Blir tröstad av kända föremål, framför allt favoritfilt eller gosedjur
**4. Förmedlar barnet sina viljor och behov?**	Barnet gråter vid hunger eller om hen är obekväm	Barnet gråter vid hunger eller om hen är obekväm	Barnet gråter vid hunger eller om hen är obekväm	Barnet gråter vid hunger eller om hen är obekväm	Vokaliserar/uttrycker behov som till exempel hunger, obehag, nyfikenhet för föremål eller omgivning	Använder enkla ord eller tecken	3-4ords meningar eller tecken<br>Kan indikera toalettbehov, kallar sig för själv eller mig
**5. Är barnet rastlöst?**	Ingen ihållande tid av alert vakenhet	Inget ihållande lugnt tillstånd	Inget ihållande lugnt tillstånd	Inget ihållande lugnt tillstånd	Inget ihållande lugnt tillstånd	Inget ihållande lugnt tillstånd	Inget ihållande lugnt tillstånd
**6. Är barnet otröstligt?**	Blir inte lugnad av att förälder vagga, sjunger, matar eller tröstar	Blir inte lugnad av att föräldern vaggar, sjunger, matar eller tröstar	Blir inte lugnad av att föräldern vaggar, sjunger, matar eller tröstar	Blir inte lugnad av att föräldern vaggar, sjunger, matar eller tröstar	Blir inte lugnad av vanliga metoder som att sjunga, hålla om, prata	Blir inte lugnad av vanliga metoder som att sjunga, hålla om, prata eller läsa	Blir inte lugnad av vanliga metoder som att sjunga, hålla om, prata eller läsa (kan få utbrott men klarar av att samla sig)
**7. Är barnet passivt – rör sig väldigt lite när han/hon är vaken?**	Lite, om ens någon tid av omväxlande spänd och avslappnad i kroppen samt primitiva reflexer (barnet borde sova lugnt den största delen av tiden)	Lite, om ens någon aktivitet som att sparka med benen, sträcka sig efter och greppa föremål (fortsatt till viss del o-koordinerat)	Lite, om ens någon aktivitet som att sparka med benen, sträcka sig efter och greppa föremål (nu möjligen mer koordinerat)	Lite, om ens något avsiktligt gripande, kontroll av huvud och arm som till exempel skjuta bort irriterande föremål	Lite, om ens något avsiktligt gripande, rörelse i sängen, skjuta bort föremål	Lite, om ens någon lek, försök att sitta upp, dra sig upp och om rörlig krypa eller gå omkring	Lite, om ens några försök till utvecklad lek, sitta upp och röra sig, och om barnet kan stå, gå eller hoppa
**8. Tar det lång tid för barnet att reagera på interaktion?**	Gör inga ljud eller aktiva reflexer som förväntat (greppa, suga, moro-reflexer)	Gör inga ljud eller aktiva reflexer som förväntat (greppa, suga, moro-reflexer)	Sparkar eller gråter inte vid procedurer som är obehagliga	Inga läten, leenden eller fokusering med blicken som svar på interaktion	Inga ljud/skratt i sociala interaktioner (eller ens försök att aktivt avvisa interaktion)	Följer inte enkla uppmaningar. Om verbal, engagerar sig inte i en enkel dialog genom ord eller jargong	Följer inte enkla 1-2stegs uppmaningar.<br>Om verbal, engagerar sig inte i lite mer komplex dialog

Additional file 2: All versions of CAPD during the translation and cultural adaptation process

Translated version by translator 2

Vänligen besvara följande frågor baserat på dina interaktioner med patienten under loppet av ditt skift:						
	Aldrig<br>4	Sällan<br>3	Ibland<br>2	Ofta<br>1	Alltid<br>0	Poäng
1. Tar barnet ögonkontakt med vårdaren?						
2. Är barnets handlingar målmedvetna?						
3. Är barnet medvetet om sin omgivning?						
4. Kommunicerar barnet sina behov och önskemål?						
	Aldrig<br>4	Sällan<br>3	Ibland<br>2	Ofta<br>1	Alltid<br>0	
5. Är barnet rastlöst?						
6. Är barnet otröstligt?						
7. Är barnet underaktivt – mycket lite rörelse när det är vaket?						
8. Dröjer det länge för barnet att svara på interaktioner?						
					TOTALT	

Utvecklingsmässiga ankarpunkter för det yngsta patienterna

	Nyfödd	4 veckor	6 veckor	8 veckor	28 veckor	1 år	2 år
**1. Tar barnet ögonkontakt med vårdaren?**	Fixerar blicken på ansiktet	Håller kort ögonkontakt<br><br>Följer 90 grader	Håller ögonkontakt	Följer rörligt föremål/vårdare förbi mittlinjen, betraktar undersökarens hand som håller ett föremål, fokuserad uppmärksamhet	Håller ögonkontakt. Föredrar den primära föräldern. Tittar på den som talar	Håller ögonkontakt. Föredrar den primära föräldern. Tittar på den som talar	Håller ögonkontakt. Föredrar den primära föräldern. Tittar på den som talar
**2. Är barnets handlingar målmedvetna?**	Vänder huvudet åt sidan, dominerad av primitiva reflexer	Sträcker sig mot något<br>(med viss brist på koordinering)	Sträcker sig mot något	Symmetriska rörelser, håller passivt fast i ett överlämnat föremål	Sträcker sig mot något med en koordinerad och jämn rörelse	Sträcker sig mot och manipulerar föremål, försöker ändra ställning, kan – om rörlig – försöka resa sig	Sträcker sig mot och manipulerar föremål, försöker ändra ställning, kan – om rörlig – försöka resa sig och gå
**3. Är barnet medvetet om sin omgivning?**	Lugna vakenperioder	Alerta vakenperioder<br><br>Vänder sig mot primärvårdarens röst<br>Kan vända sig mot primärvårdarens lukt	Alerta vakentiden ökar<br><br>Vänder sig mot primärvårdarens röst<br>Kan vända sig mot primärvårdarens lukt	Ansiktet lyser upp eller ler som svar på nickande huvud, rynkar pannan åt ringklocka, gurglar	Stark preferens för mamman, därefter andra välkända personer.<br>Skiljer mellan nya och välkända föremål	Preferens för primära föräldern, därefter andra välkända personer, ledsen om separerad från föredragna vårdare. Tröstas av välkända föremål i synnerhet älsklingsfilt eller gosedjur	Preferens för primära föräldern, därefter andra välkända personer, ledsen om separerad från föredragna vårdare. Tröstas av välkända föremål i synnerhet älsklingsfilt eller gosedjur
**4. Kommunicerar barnet sina behov och önskemål?**	Skriker vid hunger eller obehag	Skriker vid hunger eller obehag	Skriker vid hunger eller obehag	Skriker vid hunger eller obehag	Jollrar /markerar behov, t.ex. hunger, obehag, nyfikenhet om föremål eller omgivning	Använder enstaka ord eller tecken	Meningar med 3–4 ord, eller tecken. Kan markera toalettbehov, säger själv eller jag
**5. Är barnet rastlöst?**	Ingen ihållande alert vakenperiod	Inget ihållande lugnt tillstånd	Inget ihållande lugnt tillstånd	Inget ihållande lugnt tillstånd	Inget ihållande lugnt tillstånd	Inget ihållande lugnt tillstånd	Inget ihållande lugnt tillstånd
**6. Är barnet otröstligt?**	Lugnas inte av att en förälder vaggar, sjunger, matar, tröstar	Lugnas inte av att en förälder vaggar, sjunger, matar, tröstar	Lugnas inte av att en förälder vaggar, sjunger, matar, tröstar	Lugnas inte av att en förälder vaggar, sjunger, matar, tröstar	Lugnas inte med vanliga metoder, t.ex. sjunga, hålla, prata	Lugnas inte med vanliga metoder, t.ex. sjunga, hålla, prata, läsa	Lugnas inte med vanliga metoder, t.ex. sjunga, hålla, prata, läsa (Kan bli rasande men kan samla sig)
**7. Är barnet underaktivt – mycket lite rörelse när det är vaket?**	Lite eller inget spänt och sedan avslappat tillstånd med primitiva reflexer<br><br>(Barnet bör sova lugnt större delen av tiden)	Lite eller inget sträckande mot mål, sparkande, gripande (kan fortfarande vara något okoordinerat)	Lite eller inget sträckande mot mål, sparkande, gripande (kan börja bli mer koordinerat)	Lite eller inget målinriktat gripande, kontroll över huvud- och armrörelser, såsom att knuffa bort otrevliga saker	Lite eller inget sträckande mot mål, gripande, förflyttningar i sängen, bortknuffande av saker	Få eller inga lekar, försök att sätta sig upp, dra sig upp och – om rörlig – krypa eller gå omkring	Få eller inga mer utvecklade lekar, försök att sätta sig upp och röra på sig och – om i stånd att göra det – stå, gå, eller hoppa
**8. Dröjer det länge för barnet att svara på interaktioner?**	Inga ljud eller förväntade reflexer (gripa, suga, mororeflex)	Inga ljud eller förväntade reflexer (gipa, suga, mororeflex)	Sparkar eller skriker inte vid otrevlig stimulans	Inget gurglande, leende eller fokuserad ögonkontakt som svar på interaktioner	Inget joller eller leende/skratt vid sociala interaktioner (eller ens aktivt avvisande av en interaktion)	Följer inte enkla instruktioner. Om talkunnig, deltar inte i enkel dialog med ord eller nonsensord	Följer inte enkla uppmaningar med 1–2 steg. Om talkunnig, deltar inte i mer komplex dialog

copyright Cornell University 2013; alla rättigheter förbehållna

Additional file 2: All versions of CAPD during the translation and cultural adaptation process

Step 3: Reconciled version

Vänligen svara på följande frågor baserat på din interaktion med patienten under ditt arbetspass:

	Aldrig<br>4	Sällan<br>3	Ibland<br>2	Ofta<br>1	Alltid<br>0	Poäng
**1. Tar barnet ögonkontakt med vårdaren?**						
**2. Är barnets handlingar målmedvetna?**						
**3. Är barnet medvetet om sin omgivning?**						
**4. Kommunicerar barnet sina behov och önskemål?**						
	**Aldrig<br>0**	**Sällan<br>1**	**Ibland<br>2**	**Ofta<br>3**	**Alltid<br>4**	
**5. Är barnet rastlöst?**						
**6. Är barnet otröstligt?**						
**7. Är barnet passivt – mycket lite rörelse när det är vaket?**						
**8. Dröjer det länge för barnet att reagera på interaktioner?**						
					**Totalt**	

Utvecklingsnivåer utifrån ålder för de yngsta patienterna

	Nyfödda	4 veckor	6 veckor	8 veckor	28 veckor	1 år	2 år
**1. Tar barnet ögonkontakt med vårdaren?**	Fixerar blicken på ansiktet	Håller kort ögonkontakt<br>Följer 90 grader	Håller ögonkontakt	Följer rörligt föremål/vårdare förbi mittlinjen, betraktar undersökares hand som håller ett föremål, fokuserad uppmärksamhet	Håller ögonkontakt.<br>Föredrar huvudsaklig vårdare<br>Tittar på den som talar	Håller ögonkontakt.<br>Föredrar huvudsaklig vårdare<br>Tittar på den som talar	Håller ögonkontakt.<br>Föredrar huvudsaklig vårdare<br>Tittar på den som talar
**2. Är barnets handlingar målmedvetna?**	Vänder huvudet åt sidan, dominerad av primitiva reflexer	Sträcker sig mot något<br>(med viss brist på koordinering)	Sträcker sig mot något	Symmetriska rörelser, kommer passivt greppa ett överlämnat föremål	Sträcker sig mot något med en koordinerad och jämn rörelse	Sträcker sig mot och manipulerar föremål, försöker ändra ställning, kan – om rörlig – försöka resa sig	Sträcker sig mot och manipulerar föremål, försöker ändra ställning, kan – om rörlig – försöka resa sig och gå
**3. Är barnet medvetet om sin omgivning?**	Lugna vakenperioder	Alerta vakenperioder<br>Vänder sig mot huvudsaklig vårdares röst. Kan vända sig mot huvudsaklig vårdares lukt	Alerta vakenperioder<br>Vänder sig mot huvudsaklig vårdares röst. Kan vända sig mot huvudsaklig vårdares lukt	Ansiktet lyser upp eller ler som svar på nickande huvud, rynkar pannan vid starka ljud, gör uppskattande läten	Föredrar starkt huvudsaklig vårdare, därefter andra välkända personer.<br>Skiljer mellan nya och bekanta föremål	Föredrar huvudsaklig vårdare, därefter andra välkända personer, upprörd om separerad från föredragen vårdare.<br>Tröstas av välkända föremål i synnerhet älsklingsfilt eller gosedjur	Föredrar huvudsaklig vårdare, därefter andra välkända personer, upprörd om separerad från föredragen vårdare.<br>Tröstas av välkända föremål i synnerhet älsklingsfilt eller gosedjur
**4. Kommunicerar barnet sina behov och önskemål?**	Skriker vid hunger eller obehag	Skriker vid hunger eller obehag	Skriker vid hunger eller obehag	Skriker vid hunger eller obehag	Jollrar/markerar behov, t.ex. hunger, obehag, nyfikenhet för föremål eller omgivning	Använder enstaka ord eller tecken	Meningar med 3–4 ord, eller tecken. Kan markera toalettbehov, säger själv eller jag
**5. Är barnet rastlöst?**	Ingen ihållande alert vakenperiod	Inget ihållande lugnt tillstånd	Inget ihållande lugnt tillstånd	Inget ihållande lugnt tillstånd	Inget ihållande lugnt tillstånd	Inget ihållande lugnt tillstånd	Inget ihållande lugnt tillstånd
**6. Är barnet otröstligt?**	Lugnas inte av att en vårdare vaggar, sjunger, matar, tröstar	Lugnas inte av att en vårdare vaggar, sjunger, matar, tröstar	Lugnas inte av att en vårdare vaggar, sjunger, matar, tröstar	Lugnas inte av att en vårdare vaggar, sjunger, matar, tröstar	Lugnas inte med vanliga metoder, t.ex. sjunga, hålla, prata	Lugnas inte med vanliga metoder, t.ex. sjunga, hålla, prata, läsa	Lugnas inte med vanliga metoder, t.ex. sjunga, hålla, prata, läsa (Kan bli rasande men kan samla sig)
**7. Är barnet passivt – mycket lite rörelse när det är vaket?**	Lite eller inget flexorläge (ihopkrupen ställning) och sedan avslappnat tillstånd med primitiva reflexer<br>(Barnet bör sova lugnt större delen av tiden)	Lite eller inget sträckande mot mål, sparkande, gripande (kan fortfarande vara något okoordinerat)	Lite eller inget sträckande mot mål, sparkande, gripande (kan börja bli mer koordinerat)	Lite eller inget målinriktat gripande, kontroll över huvud- och armrörelser, såsom att knuffa bort irriterande föremål	Lite eller inget sträckande mot mål, gripande, förflyttningar i sängen, bortknuffande av föremål	Få eller inga lekar eller försök att sätta sig upp, dra sig upp och –om rörlig – krypa eller gå omkring	Få eller inga mer utvecklade lekar eller försök att sätta sig upp och röra på sig och – om i stånd att göra det – stå, gå, eller hoppa
**8. Dröjer det länge för barnet att reagera på interaktioner?**	Inga ljud eller förväntade reflexer (gripa, suga, mororeflex)	Inga ljud eller förväntade reflexer (gripa, suga, mororeflex))	Sparkar eller skriker inte vid obehagligt stimuli	Inga uppskattande läten, leende eller fokuserad ögonkontakt som svar på interaktioner	Inget joller eller leende/skratt vid sociala interaktioner (eller ens aktivt avvisande av en interaktion)	Följer inte enkla instruktioner. Om talkunnig, deltar inte i enkel dialog med ord eller nonsensord	Följer inte enkla uppmaningar med 1–2 steg. Om talkunnig, deltar inte i mer komplex dialog

Additional file 2: All versions of CAPD during the translation and cultural adaptation process

Step 4: Back-translated version

Please answer the following questions based on your interaction with the patient during your shift:

	Never<br>4	Rarely<br>3	Sometimes<br>2	Often<br>1	Always<br>0	Points
**1. Does the child make eye contact with the caregiver?**						
**2. Are the child's actions purposeful?**						
**3. Is the child aware of their surroundings?**						
**4. Does the child communicate their needs and wishes?**						
	**Never<br>0**	**Rarely<br>1**	**Sometimes<br>2**	**Often<br>3**	**Always<br>4**	
**5. Is the child restless?**						
**6. Is the child inconsolable?**						
**7. Is the child passive – very little movement when awake?**						
**8. Does it take the child a long time to react to interactions?**						
					**Total**	

Cornell Assessment of Pediatric Delirium (CAPD) Re-translated version

Developmental anchoring points for the youngest patients.

	Newborn	4 weeks	6 weeks	8 weeks	28 weeks	1 year	2 years
**1. Does the child make eye contact with the caregiver?**	Fixes gaze on the face	Briefly maintains eye contact<br>Follows 90 degrees	Maintains eye contact	Follows moving objects/caregiver past the midline, watches the examiner's hand which holds an object, focused attention	Maintains eye contact. Prefers main caregiver<br>Looks at the person speaking	Maintains eye contact. Prefers main caregiver<br>Looks at the person speaking	Maintains eye contact.<br>Prefers main caregiver<br>Looks at the person speaking
**2. Are the child's actions purposeful?**	Turns head to the side, dominated by primitive reflexes	Reaches<br>toward something (with some lack of coordination)	Reaches<br>toward something	Symmetrical moments, will passively<br>grasp a handed-over object	Reaches<br>toward something with a coordinated and even movement	Reaches<br>toward and manipulates objects, tries to change position, can – if mobile – try to stand	Reaches toward and manipulates objects, tries to change position, can – if mobile – try to stand and walk
**3. Is the child aware of their surroundings?**	Calm periods awake	Alert periods awake<br>Turns toward main caregiver's voice. Can turn toward<br>main caregiver's scent	Alert periods awake<br>Turns toward main caregiver's voice. Can turn toward<br>main caregiver's scent	Face lights up or smiles in response to nodding head, wrinkles forehead during loud sounds, makes appreciative noises	Strongly prefers main caregiver, followed by other well-known people.<br>Differentiates between new and familiar objects	Prefers main caregiver, followed by other well-known people, upset if separated from main caregiver.<br>Comforted by familiar objects, especially a favorite blanket or soft toy	Prefers main caregiver, followed by other well-known people, upset if separated from main caregiver. Comforted by familiar objects, especially a favorite blanket or soft toy
**4. Does the child communicate their needs and wishes?**	Screams with hunger or discomfort	Screams with hunger or discomfort	Screams with hunger or discomfort	Screams with hunger or discomfort	Babbles/indicates needs, such as hunger, discomfort, curiosity about objects or surroundings	Uses individual words or signs	Sentences with 3–4 words, or signs. Can indicate need for toilet, says "self" or "I"
**5. Is the child restless?**	No prolonged alert period awake	No prolonged calm state	No prolonged calm state	No prolonged calm state	No prolonged calm state	No prolonged calm state	No prolonged calm state
**6. Is the child inconsolable?**	Is not calmed by caregiver rocking, singing, feeding, comforting	Is not calmed by caregiver rocking, singing, feeding, comforting	Is not calmed by caregiver rocking, singing, feeding, comforting	Is not calmed by caregiver rocking, singing, feeding, comforting	Is not calmed by usual methods, such as singing, holding, talking	Is not calmed by usual methods, such as singing, holding, talking, reading	Is not calmed by usual methods, such as singing, holding, talking, reading (May become furious, but can collect themself)
**7. Is the child passive – very little movement when awake?**	Little or no flexor position (curled-up position) and then relaxed state with primitive reflexes<br>(The child should sleep calmly much of the time)	Little or no reaching toward objects, kicking, grasping (may still be somewhat uncoordinated)	Little or no reaching toward objects, kicking, grasping (may begin to be more coordinated)	Little or no purposeful grasping, control of head and arm movements, such as pushing away annoying objects	Little or no reaching toward objects, grasping, moving in bed, pushing away of objects	Few or no games or attempts to sit up, pull themselves up and – if mobile – crawl or walk around	Few or no more developed games or attempts to sit up and move and – if able to do so – stand, walk or jump
**8. Does it take the child a long time to react to interactions?**	No sounds or expected reflexes (grasping, sucking, Moro reflex)	No sounds or expected reflexes (grasping, sucking, Moro reflex)	Does not kick or scream in response to unpleasant stimuli	No appreciative sounds, smiling or focused eye contact in response to interactions	No babbling or smiling/laughing in response to social interactions (or even active rejection of an interaction)	Does not follow simple instructions. If able to speak, does not take part in simple dialogue with words or nonsense words	Does not follow simple challenges with 1–2 steps. If able to speak, does not take part in more complex dialogue

Cornell Assessment of Pediatric Delirium (CAPD) Re-translated version

Additional file 2: All versions of CAPD during the translation and cultural adaptation process

Step 6: Harmonization

Vänligen svara på följande frågor baserat på din interaktion med patienten under ditt arbetspass:

	Aldrig<br>4	Sällan<br>3	Ibland<br>2	Ofta<br>1	Alltid<br>0	Poäng
**1. Tar barnet ögonkontakt med vårdaren?**						
**2. Är barnets handlingar målmedvetna?**						
**3. Är barnet medvetet om sin omgivning?**						
**4. Kommunicerar barnet sina behov och önskemål?**						
	**Aldrig<br>0**	**Sällan<br>1**	**Ibland<br>2**	**Ofta<br>3**	**Alltid<br>4**	
**5. Är barnet rastlöst?**						
**6. Är barnet otröstligt?**						
**7. Är barnet passivt – mycket lite rörelse när det är vaket?**						
**8. Dröjer det länge för barnet att reagera på interaktioner?**						
					**Totalt**	

Cornell Assessment of Pediatric Delirium (CAPD) Svenska version

Utvecklingsmässiga förankringspunkter för de yngsta patienterna.

	Nyfödda	4 veckor	6 veckor	8 veckor	28 veckor	1 år	2 år
**1. Tar barnet ögonkontakt med vårdaren?**	Fixerar blicken på ansiktet	Håller kort ögonkontakt<br>Följer 90 grader	Håller ögonkontakt	Följer rörligt föremål/vårdare förbi mittlinjen, betraktar undersökares hand som håller ett föremål, fokuserad uppmärksamhet	Håller ögonkontakt.<br>Föredrar huvudsaklig vårdare<br>Tittar på den som talar	Håller ögonkontakt.<br>Föredrar huvudsaklig vårdare<br>Tittar på den som talar	Håller ögonkontakt.<br>Föredrar huvudsaklig vårdare<br>Tittar på den som talar
**2. Är barnets handlingar målmedvetna?**	Vänder huvudet åt sidan, dominerad av primitiva reflexer	Sträcker sig mot något<br>(med viss brist på koordinering)	Sträcker sig mot något	Symmetriska rörelser, kommer passivt greppa ett överlämnat föremål	Sträcker sig mot något med en koordinerad och jämn rörelse	Sträcker sig mot och manipulerar föremål, försöker ändra ställning, kan – om rörlig – försöka resa sig	Sträcker sig mot och manipulerar föremål, försöker ändra ställning, kan – om rörlig – försöka resa sig och gå
**3. Är barnet medvetet om sin omgivning?**	Lugna vakenperioder	Alerta vakenperioder<br>Vänder sig mot huvudsaklig vårdares röst. Kan vända sig mot huvudsaklig vårdares lukt	Alerta vakenperioder<br>Vänder sig mot huvudsaklig vårdares röst. Kan vända sig mot huvudsaklig vårdares lukt	Ansiktet lyser upp eller ler som svar på nickande huvud, rynkar pannan vid starka ljud, gör uppskattande läten	Föredrar starkt huvudsaklig vårdare, därefter andra välkända personer.<br>Skiljer mellan nya och bekanta föremål	Föredrar huvudsaklig vårdare, därefter andra välkända personer, upprörd om separerad från föredragen vårdare.<br>Tröstas av välkända föremål i synnerhet älsklingsfilt eller gosedjur	Föredrar huvudsaklig vårdare, därefter andra välkända personer, upprörd om separerad från föredragen vårdare.<br>Tröstas av välkända föremål i synnerhet älsklingsfilt eller gosedjur
**4. Kommunicerar barnet sina behov och önskemål?**	Skriker vid hunger eller obehag	Skriker vid hunger eller obehag	Skriker vid hunger eller obehag	Skriker vid hunger eller obehag	Jollrar/markerar behov, t.ex. hunger, obehag, nyfikenhet för föremål eller omgivning	Använder enstaka ord eller tecken	Meningar med 3–4 ord, eller tecken. Kan markera toalettbehov, säger själv eller jag
**5. Är barnet rastlöst?**	Ingen ihållande alert vakenperiod	Inget ihållande lugnt tillstånd	Inget ihållande lugnt tillstånd	Inget ihållande lugnt tillstånd	Inget ihållande lugnt tillstånd	Inget ihållande lugnt tillstånd	Inget ihållande lugnt tillstånd
**6. Är barnet otröstligt?**	Lugnas inte av att en vårdare vaggar, sjunger, matar, tröstar	Lugnas inte av att en vårdare vaggar, sjunger, matar, tröstar	Lugnas inte av att en vårdare vaggar, sjunger, matar, tröstar	Lugnas inte av att en vårdare vaggar, sjunger, matar, tröstar	Lugnas inte med vanliga metoder, t.ex. sjunga, hålla, prata	Lugnas inte med vanliga metoder, t.ex. sjunga, hålla, prata, läsa	Lugnas inte med vanliga metoder, t.ex. sjunga, hålla, prata, läsa (Kan bli rasande men kan samla sig)
**7. Är barnet passivt – mycket lite rörelse när det är vaket?**	Lite eller inget flexorläge (ihopkrupen ställning) och sedan avslappnat tillstånd med primitiva reflexer<br>(Barnet bör sova lugnt större delen av tiden)	Lite eller inget sträckande mot mål, sparkande, gripande (kan fortfarande vara något okoordinerat)	Lite eller inget sträckande mot mål, sparkande, gripande (kan börja bli mer koordinerat)	Lite eller inget målinriktat gripande, kontroll över huvud- och armrörelser, såsom att knuffa bort irriterande föremål	Lite eller inget sträckande mot mål, gripande, förflyttningar i sängen, bortknuffande av föremål	Få eller inga lekar eller försök att sätta sig upp, dra sig upp och –om rörlig – krypa eller gå omkring	Få eller inga mer utvecklade lekar eller försök att sätta sig upp och röra på sig och – om i stånd att göra det – stå, gå, eller hoppa
**8. Dröjer det länge för barnet att reagera på interaktioner?**	Inga ljud eller förväntade reflexer (gripa, suga, mororeflex)	Inga ljud eller förväntade reflexer (gripa, suga, mororeflex))	Sparkar eller skriker inte vid obehagligt stimuli	Inga uppskattande läten, leende eller fokuserad ögonkontakt som svar på interaktioner	Inget joller eller leende/skratt vid sociala interaktioner (eller ens aktivt avvisande av en interaktion)	Följer inte enkla instruktioner. Om talkunnig, deltar inte i enkel dialog med ord eller nonsensord	Följer inte enkla uppmaningar med 1–2 steg. Om talkunnig, deltar inte i mer komplex dialog

Cornell Assessment of Pediatric Delirium (CAPD) Svenska version
